# Supplementary material for: Learning curve and surgical outcome of robotic assisted colorectal surgery with ERAS program
Source: Sci Rep. 2022 Nov 29;12:20566. doi: 10.1038/s41598-022-24665-w (PMC9709162; doi:10.1038/s41598-022-24665-w)
Supplement: Supplementary file 1 — Supplementary Information 1. [file 41598_2022_24665_MOESM1_ESM.zip › LSC Raw data-Ñ[▒K/084/O_ñw╝╨░O▒Kñσ.pdf]

正式摘要(電子簽章：00000194819788)

W52 / W52

臺中榮民總醫院

Operation Note

[redacted]

- 手術日期：2020/07/16
- 手術醫師：VS 蔣鋒帆 , CR 楊曙榮 , R 陳凱祥
- Anesthesia : GE
- Position : Lithotomy
- Aseptic agents : Soap , Betadine Alcohol , Betadine Aqua

Diagnosis

- Pre-operative diagnosis : CA OF PROXIMAL S COLON
- Post-operative diagnosis : Ditto.

Surgical Indication

- Tumor resection

Operation

- 1. Laparoscopic anterior resection with side-to-end anastomosis

Finding

- 1. One 4x6 cm tumor located at proximal sigmoid colon
  2. There was no palpable liver metastasis, no visible peritoneal seeding.
  3. High ligation (-) below the left colic artery.
  4. Take down splenic flexure (+)
  5. Drain: 12 penrose in pelvis, hyalobarrier+

Procedure

- Under GE, the patient was put in lithotomy position. 5 troca hole was made, the first was by open method. Pneumoperitonium was created. the Olympus Endo-EYE laparoscope was inserted from umbilical port. Abdominal exploration was done and the findings were mentioned above. The patient position was adjusted to head-down and right tilting to let small bowels move to RUQ and the pelvis was well exposured. The IMV was identified, dissected, skeletonized by Harmonic scalpel and transected by touble clips and Harmonic energy device. The left colic artery was done in the same way. The mobilization of S-colon was done with dissection via embryonic plane between Gerota fascia and meso-sigmoid colon. The lateral attachement of sigmoid colon was also divided. The IMA finally was dissected and transected by touble clips and Harmonic energy device. The slpenic flexure was take down. After fully mobilization of S-colon, transection of S-colon was done by EndoGIA 60/3.2mm endocutters. The speciemn was removed from periumbilical wound . The 29 mm envil was inserted in proximal colon annd take back into abdominal cavity. The side to end colorectal anastomosis was done by double stappling technique. After air-leak test, hyalobarrier was left on medline wound. Penrosce drain was placed to the pelvic from the LLQ troca. 4 ports and was closed in layers. Throughout the whole course of operation, patient stood quite well.
- Estimated blood loss: minimum
- 器械數量: 正確
- 紗布數量: 正確

Image

- PAINT
  - abdomen

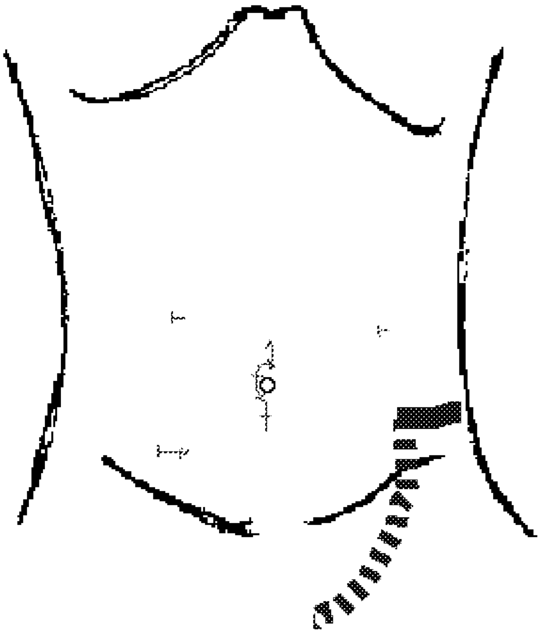

○ colon & rectum

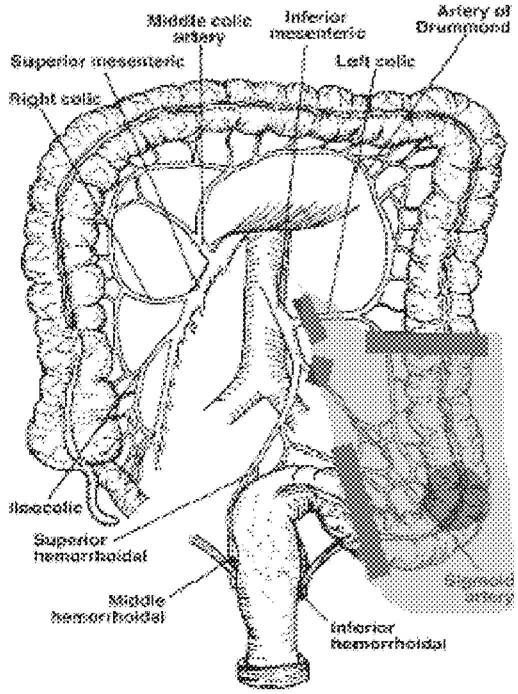

主任 2062F蔣鋒帆 / 醫師 2292G楊曙榮

正式摘要(**電子簽章：000000194819788)**

W52 / W52

doctorRank:B
